# Comprehensive characterization of pre- and post-treatment samples of breast cancer reveal potential mechanisms of chemotherapy resistance

**DOI:** 10.1038/s41523-022-00428-8

**Published:** 2022-05-06

**Authors:** Marlous Hoogstraat, Esther H. Lips, Isabel Mayayo-Peralta, Lennart Mulder, Petra Kristel, Ingrid van der Heijden, Stefano Annunziato, Maartje van Seijen, Petra M. Nederlof, Gabe S. Sonke, Wilbert Zwart, Jelle Wesseling, Lodewyk F. A. Wessels

**Affiliations:** 1 Division of Oncogenomics, Oncode Institute, The Netherlands Cancer Institute, Amsterdam, The Netherlands; 2 Division of Molecular Carcinogenesis, Oncode Institute, The Netherlands Cancer Institute, Amsterdam, The Netherlands; 3 Division of Molecular Pathology, The Netherlands Cancer Institute, Amsterdam, the Netherlands; 4 Department of Pathology, The Netherlands Cancer Institute, Amsterdam, The Netherlands; 5 Division of Medical Oncology, Netherlands Cancer Institute, Amsterdam, The Netherlands; 6 Department of Pathology, Leiden University Medical Center, Leiden, The Netherlands; 7 Department of EEMCS, Delft University of Technology, Delft, The Netherlands

**Keywords:** Breast cancer, Breast cancer, Tumour biomarkers, RNA sequencing

## Abstract

When locally advanced breast cancer is treated with neoadjuvant chemotherapy, the recurrence risk is significantly higher if no complete pathologic response is achieved. Identification of the underlying resistance mechanisms is essential to select treatments with maximal efficacy and minimal toxicity. Here we employed gene expression profiles derived from 317 HER2-negative treatment-naïve breast cancer biopsies of patients who underwent neoadjuvant chemotherapy, deep whole exome, and RNA-sequencing profiles of 22 matched pre- and post-treatment tumors, and treatment outcome data to identify biomarkers of response and resistance mechanisms. Molecular profiling of treatment-naïve breast cancer samples revealed that expression levels of proliferation, immune response, and extracellular matrix (ECM) organization combined predict response to chemotherapy. Triple negative patients with high proliferation, high immune response and low ECM expression had a significantly better treatment response and survival benefit (HR 0.29, 95% CI 0.10–0.85; *p* = 0.02), while in ER+ patients the opposite was seen (HR 4.73, 95% CI 1.51–14.8; *p* = 0.008). The characterization of paired pre-and post-treatment samples revealed that aberrations of known cancer genes were either only present in the pre-treatment sample (*CDKN1B*) or in the post-treatment sample (*TP53, APC, CTNNB1*). Proliferation-associated genes were frequently down-regulated in post-treatment ER+ tumors, but not in triple negative tumors. Genes involved in ECM were upregulated in the majority of post-chemotherapy samples. Genomic and transcriptomic differences between pre- and post-chemotherapy samples are common and may reveal potential mechanisms of therapy resistance. Our results show a wide range of distinct, but related mechanisms, with a prominent role for proliferation- and ECM-related genes.

## Introduction

Chemotherapy is currently the standard of care for primary breast cancer with high risk of recurrence and can be administered in an adjuvant or neoadjuvant setting. Neoadjuvant administration increases the chances of breast- and axilla-conserving surgery by downstaging of the tumor^1^. Moreover, it allows in vivo assessment of the tumor’s response to the treatment and can therefore help to more accurately determine a patient’s prognosis and guide adjuvant treatment. If a pathological complete response (pCR) is achieved, the prognosis is good, especially in high-grade and triple negative tumors^2^. In cases of partial or non-response, however, recurrences are frequent^3^.

Many studies have focused on the development of biomarkers to predict response to chemotherapy in the neoadjuvant setting, using pCR as primary outcome measure. Most studies report on the predictive value of proliferation, but activation of the immune system is also recurrently identified as a predictive factor^4–7^. It should be noted that chemotherapy does not induce ‘all or nothing’ responses. Rather, the vast majority of tumors shows a partial, but incomplete, response. Methods to further characterize response to neoadjuvant chemotherapy such as the Neoadjuvant Response Index (NRI)^8^ and Residual Breast Cancer Burden (RCB) index^9^ have shown that the extent of non-response is also predictive of recurrence-free survival.

Importantly, a partial response to treatment suggests that only part of the tumor may be resistant to treatment, or that a resistance mechanism has appeared under treatment pressure. A sample taken before treatment may thus contain both treatment sensitive and resistant cells, which impedes the discovery of biomarkers for treatment response. Studies that compared samples obtained after treatment to pre-treatment samples have shown considerable successes in revealing resistance mechanisms to various targeted cancer drugs^10–13^. However, the success rates have been more limited for identifying such mechanisms to different chemotherapy regimens^14,15^. Here, we performed both RNA sequencing of a large sample set of pre-treatment biopsies, as well as deep DNA and RNA characterization of paired pre- and post-treatment samples. In addition, we used a more quantitative response measurement, the NRI, as primary outcome method. By analyzing both treatment naïve and post treatment samples, as well as assessing response precisely, we aimed to identify biomarkers for response and decipher mechanisms of therapy resistance.

## Results

### Overview patient cohort and therapy response

Expression data was collected from pre-treatment biopsies of 317 HER2-negative breast cancer patients, all treated with neoadjuvant chemotherapy between 2000 and 2013. Both ER-positive (ER+, *n* = 200) and ER-negative (‘triple negative, TN’, *n* = 117) patients were included (Table 1). The majority of the patients were treated with 6 courses of dose dense doxorubicin and cyclophosphamide (ddAC, *n* = 186), or 3 courses of ddAC and 3 courses of capecitabine and docetaxel (CD) (*n* = 49). Pathological complete response (pCR; the absence of invasive tumor cells in the breast and axilla) was used as primary measure of response to chemotherapy, which could be determined for 301 patients. Of the ER+ group, only 3.5% (*n* = 7) achieved a pCR. For TN, this was 39% (*n* = 46). Long-term follow-up was collected with a median follow-up of 6.2 years. In TN patients, pCR was associated with improved survival (*p* = 0.0056, HR = 0.223 (95% CI: 0.077–0.645)) (Supplementary Fig. 1). In ER+ patients, the same trend was observed but it did not reach significance (*p* = 0.997, HR = 3.89e−8 (95% CI: 0—inf) (Supplementary Fig. 2). The Neoadjuvant Response Index (NRI), an alternative, semi-continuous measure of response^8^, was determined for 253 patients and could be used to further assess therapy response in patients who did not achieve a pCR. As described previously^16^, NRI was significantly associated with recurrence free survival in TN patients (*p* = 0.0003, HR = 0.12 (95% CI: 0.038–0.379)). NRI was significantly different between ER+ and TN patients (median NRI 0.29 versus 0.67 for ER+ and TN tumors, respectively, *p* = 2.61e−16).

**Table 1:** Patient characteristics of the full cohort of 317 patients.

		LUM (*n* = 200)	TN (*n* = 117)	LUM (100%)	TN (100%)
Age	Mean (sd)	48.7 (9.8)	44.3 (11.0)		
Grade	1	10	0	5.00	0.00
	2	102	25	51.00	21.37
	3	48	70	24.00	59.83
	Missing	40	22	20.00	18.80
T-stage	1	19	10	9.50	8.55
	2	111	74	55.50	63.25
	3	50	17	25.00	14.53
	4	12	10	6.00	8.55
	Missing	8	6	4.00	5.13
N-stage	Negative	38	39	19.00	33.33
	Positive	153	72	76.50	61.54
	Missing	9	6	4.50	5.13
pCR	No	183	65	91.50	55.56
	Yes	7	46	3.50	39.32
	Missing	10	6	5.00	5.13
Treatment	6× ddAC	117	69	58.50	58.97
	6× CD	11	1	5.50	0.85
	3× ddAC, 3× CD	39	10	19.50	8.55
	Other	25	33	12.50	28.21
	Missing	8	4	4.00	3.42

*LUM* luminal, *TN* triple negative, *ddAC* dose-dense adriamycin + cyclophosphamide, *CD* capecitabine + docetaxel.

### Proliferation rate, immune response and ECM jointly predict response to neoadjuvant chemotherapy

We then used our gene expression data to find markers of therapy response. Because the response rates between ER+ and TN samples were vastly different (Table 1), analyses to detect biomarkers of response using all samples yielded many genes associated with ER-status, potentially obscuring more subtle gene expression differences between responders and non-responders. We therefore analyzed the subtypes separately. NRI was our primary outcome measure, although we also assessed if there was an association with pCR and recurrence free survival.

Differential gene expression in ER+ samples with NRI as outcome variable did not yield many genes showing significant association with outcome: in only 48 genes, FDR rates were below 20% and functional enrichment analyses did not identify specific biological processes. However, in TN samples the same approach resulted in 778 genes positively and 826 genes negatively associated with NRI, with an FDR of <20% (Supplementary data file 1). Negatively associated genes included homeobox proteins, ABC transporters, and genes involved in extracellular matrix organization (ECM) (Supplementary data file 1). Functional enrichment of positively associated genes revealed genes predominantly associated with immune response and cell proliferation, including *CDKN2A* and several cyclins.

**Fig. 1:**
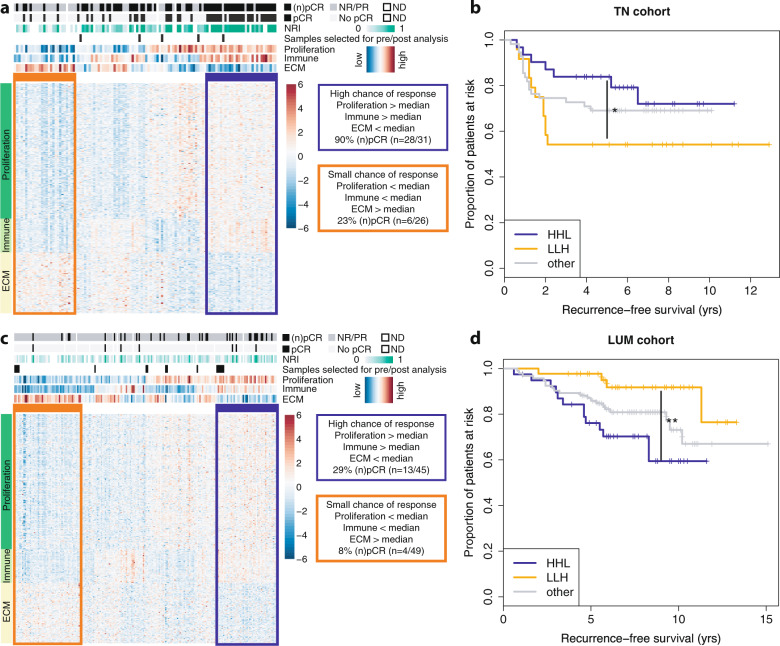
Biological processes associated with response to neoadjuvant chemotherapy. **a** Heatmap of expression levels of genes associated with response to neoadjuvant chemotherapy in triple negative breast cancer patients. Cases are in columns, genes in rows. Biological processes associated with the genes in this heatmap are indicated on the left; patient and sample information are indicated on the top: top track indicates treatment response in the breast; second and third tracks indicate treatment response in the breast and lymph nodes. The three bottom tracks indicate the average gene expression level of the genes associated with proliferation, immune response and ECM. The orange and blue boxes respectively indicate the gene expression profiles of poor and good responders. Black bars at the bottom indicate which samples were included in the pre/post chemotherapy analyses. ECM extracellular matrix, ND not determined, (n)pCR (near) pathological complete response, NRI neoadjuvant response index. **b** Kaplan–Meier curves showing recurrence-free survival in subgroups of triple negative patients as identified in (**a**). HHL (blue) High proliferation, High immune response, Low ECM, LLH (orange) Low proliferation, Low immune response, High ECM, and other combinations (gray). **c** Heatmap of expression levels of genes associated with response to neoadjuvant chemotherapy in ER+/HER2− breast cancer patients. Format is the same as in (**a**). **d** Kaplan–Meier curves showing recurrence-free survival in subgroups of ER+/HER2− patients as identified in (**c**). *p < 0.05; **p < 0.01 (Kaplan–Meier estimate).

Based on these results, we selected a ‘core enrichment’ set of genes with the strongest association with NRI (FDR < 5%). We then employed the expression levels of this core set to stratify TN patient samples based on the activity of proliferation (*n* = 176), immune response (*n* = 44) and ECM (*n* = 79) genes (see the “Methods“ section) (Fig. 1a). When overlapping our list of genes upregulated in responders with the reporter genes used in CIBERSORT^17^, we observed a significant enrichment for genes expressed in activated natural killer cells, M1 Macrophages, activated dendritic cells and CD8+ T-cells (adjusted *p* < 0.001, <0.001, <0.001 and =0.03, respectively) (Supplementary data file 1). These cell types are associated with cytolytic activity and tumor-killing potential^18^. The median expression levels of the proliferation-associated genes showed a positive correlation with Ki67 immunohistochemistry scores (*R* = 0.673, Supplementary Fig. 3), indicating that high expression levels of these genes are indicative of highly proliferative tumor cells. The highest response rates were observed for TN patients that showed high expression of both proliferation and immune response genes, and low expression of ECM genes (Proliferation = H, Immune = H, ECM = L, denoted as ‘HHL’). In contrast, patients at the other end of the spectrum (Proliferation = L, Immune = L, ECM = H, denoted as ‘LLH’) showed the lowest response rates (measured as (*n*)pCR, *p* < 0.001). In line with this observation, HHL-patients showed significantly higher 5-year recurrence-free survival rates, compared to the LLH group (*p* = 0.02, HR 0.29 (95%CI 0.10–0.85), Fig. 1b). Patients who could not be stratified in either the LLH or HHL group (denoted hereafter as ‘other’) showed intermediate response and survival rates.

To investigate the value of this stratification method in an independent cohort, we stratified basal breast cancer patient samples from the TCGA PanCancer cohort^19^ into an ‘HHL’, an ‘LLH’ and an ‘other’ group. We then assessed progression-free survival rates in the three subgroups. Using all samples, a similar trend as observed in the neoadjuvant cohort was visible, but not significant (Supplementary Fig. 4A, *p* = 0.2). We subsequently performed analyses stratified for clinical subgroups in the TCGA basal cohort, defined as high-risk (lymph-node positive (N+) or T-stage 3/4) (Supplementary Fig. 4B), lymph-node negatve versus positive (Supplementary Fig. 4C, D), and T-stage 1/2 versus T-stage 3/4 (Supplementary Fig. 4E, F). In high-risk patients and in N+ patients, HHL-patients show significantly higher progression-free survival rates compared to LLH- and other patients (*P* = 0.02 and *P* = 0.004, respectively), while in node negative and T-stage 1/ 2, no differences in survival rates were observed.

We then tested whether the genes identified in TN tumors also have predictive power in ER+ breast cancer: 62 genes from the core enrichment set were associated with (near) pCR in ER+ tumors as well (Supplementary data file 1). The majority of these genes were involved in proliferation, while genes involved in immune response or ECM did not appear to have a large effect on chemotherapy response in ER+ breast cancer. By clustering ER+ patients using the core enrichment genes identified in TN tumors (see the “Methods” section), we observed a higher NRI (*p* = 0.053) and a significantly higher (*n*)pCR rate (*p* = 0.014) for patients with an HHL expression pattern, compared to those with an LLH profile (Fig. 1c). However, in contrast to the TN patients, ER+ patients falling in the LLH group had a significantly better prognosis than patients with an HHL profile (*p* = 0.008, HR 4.73 (95% CI 1.51–14.81), Fig. 1d). For luminal-type breast cancer we could not validate our results in the luminal TCGA cohort (Supplementary Fig. 5). We have to keep in mind here that the TCGA luminal set is much more heterogenous than our neoadjuvant luminal cohort, indicating that it is difficult to cross-validate our findings.

### Decreased proliferation rates after chemotherapy are not indicative of improved survival in ER-positive tumors

While we can now predict the response of tumors with HHL and LLH profiles, the response of tumors falling outside of these profiles is still uncertain. An incomplete response to chemotherapy may in part be due to the presence or outgrowth of a resistant subclone, not necessarily detectable in a pre-treatment biopsy. To investigate therapy resistant samples further, we therefore collected matched pre- and post-treatment samples and normal blood as a germline control from 22 patients who did not achieve a pCR on neoadjuvant chemotherapy. This dataset included six triple negative (TN) tumors and sixteen ER+ ductal carcinomas (ER+) (Table 2). We specifically selected samples with a tumor cell percentage of 40% or higher, resulting in an enrichment of tumors with a substantial amount of residual disease. Even though the selection of these patients was determined by sample availability, their pre-treatment samples were representative of non-responder gene expression signatures in the full cohort (Fig. 1a, c). In all pre- and post-treatment samples, we measured gene expression levels and performed deep (>150× coverage) whole exome sequencing (WES) to identify mutations and structural variants (see Supplementary data file 2 for sequencing statistics).

**Table 2:** Characteristics of the 22 patients with pre-and post chemotherapy matched samples.

StudyID	Histology	Regimen	NRI	RCB	Grade	T-stage	N-stage	Subtype	Recurrence	Recurrence location	Vital status	Exome seq	RNA seq	DNA_pre_TP	DNA_post_TP	RNA_pre_TP	RNA_post_TP
97	IDC	6× ddAC	0.33	2	NA	2	−	LUM	0		A	1	1	70	80	70	75
188	IDC	1×AC, 1×CD	0	2	3	2	−	TN	1	Lung, brain	D	1	0	80	80		
2046	IDC	6× ddAC	0	3	3	2	+	TN	1	Liver	D	1	1	70	70	50	70
2271	IDC	6× ddAC	0.33	2	3	2	+	TN	1	Thorax, lymph, contralateral mamma, skin	D	1	1	80	55	90	55
2472	IDC	4× ddAC, 2× CTC + PSCT	0.33	3	2	2	+	TN	1	Multiple lymph nodes, lung, liver	D	0	1			80	70
2492	IDC	6× ddAC	0.33	3	2	2	+	TN	1	Brain	D	1	1	90	50	60	50
2653	IDC	3× ddAC, 2× CD, 2× D	0	2	2	2	+	LUM	1	Bone	A	0	1			60	50
2661	Papillary carcinoma	6× ddAC	0.2	NA	1	4	−	LUM	0		A	1	1	90	80	90	80
2677	ILC	6× ddAC	0	NA	2	2	−	LUM	0		A	1	1	50	70	50	70
2691	IDC	6× ddAC	0.33	3	2	2	+	LUM	0		A	1	1	40^a^	60	40^a^	60
2739	IDC	3× AC, 3× CD	0.33	2	2	2	+	LUM	0		A	1	1	80	60	80	60
2789	IDC	6× ddAC	0.33	2	2	2	−	LUM	0		A	1	1	60	40	60	40
2817	IDC	3× AC, 3× CD	0	3	2	2	+	LUM	0		A	1	1	70	80	70	80
2852	IDC	3× ddAC	0	3	2	2	+	LUM	1	Bone, liver	A	1	1	70	60	70	60
2976	IDC	3× ddAC, 4× paclitaxel	0	2	2	2	−	LUM	0		A	1	1	70	50	70	50
3001	IDC	6× ddAC	0	3	2	2	+	LUM	0		A	1	1	60	65	60	65
3036	IDC	6× ddAC	0.25	2	1	3	+	LUM	0		A	1	1	50	50	50	50
3065	IDC	6x ddAC	0	2	2	2	+	LUM	0		A	1	1	70	60	70	60
3067	ILC	6x ddAC	0	3	2	1	+	LUM	0		A	1	1	60	60	60	60
3130	IDC	6x ddAC	0	3	2	1	+	LUM	0		A	1	1	60	55	60	55
3161	IDC	3x ddAC, 9× paclitaxel	0	3	2	2	+	LUM	0		A	1	1	60	80	60	80
3280	Metaplastic	3× ddAC, 3× carbo/paclitaxel	NA	NA	3	2	+	TN	0		A	1	0	60	60		

*NRI* Neoadjuvant Response Index, *RCB* residual breast cancer burden, *IDC* invasive ductal carcinoma, *ILC* invasive lobular carcinoma, *ddAC* dose-dense adriamycin + cyclophosphamide, *CD* capecitabine + docetaxel, *CTC* *+* *PSCT* cyclofosfamide-thiotepa-carboplatine and peripheral stem cell transplant, *TN* triple negative, *LUM* luminal, *A* alive, *D* dead, *DNA_pre_TP* tissue tumor percentage pre-treatment for DNA isolation, *DNA_post_TP* tissue tumor percentage post-treatment for DNA isolation, *RNA_pre_TP* tissue tumor percentage pre-treatment for RNA isolation, *RNA_post_TP* tissue tumor percentage post-treatment for RNA isolation.

^a^Although we applied a 50% cut-off for tumor percentage, one exception was taken along.

**Fig. 2:**
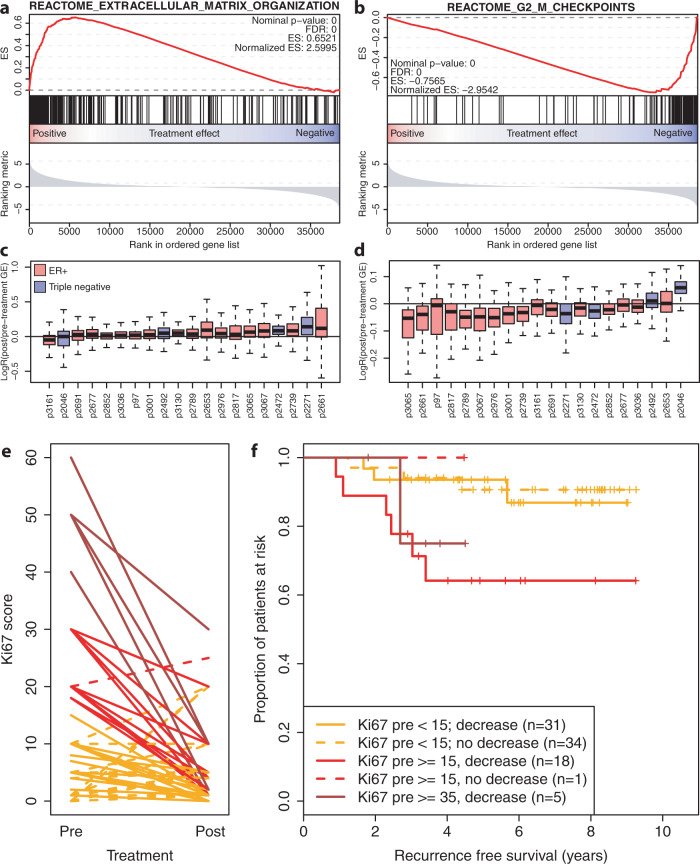
Transcriptomic differences between breast tumors before and after chemotherapy. **a** Gene set enrichment plot of genes involved in extracellular matrix organization according to the reactome database. Genes are ordered based on the differential gene expression analyses: genes on the right side of the plot are down-regulated upon treatment, whereas genes on the left are upregulated. FDR: False Discovery Rate, ES enrichment score. **b** Gene set enrichment plot of genes involved in the G-phase to M-phase checkpoint according to the reactome database. Layout is identical to (**a**). **c** Boxplots of log fold changes between pre- and post-treatment samples of the geneset shown in (**a**). Every box is one sample: red are ER+ samples, blue are triple negative. The box indicates the interquartile range, while lower and upper bars correspond to the minimum and maximum non-outlier values of the data distribution. Outliers are defined as values outside 1.5× the interquartile range from the box. The center line indicates the median value. **d** Boxplots of log fold changes between pre- and post-treatment samples of the geneset shown in (**b**). Every box is one sample: red boxes are ER+ samples, blue boxes are triple negative. Boxplots are constructed in the same manner as in (**c**). **e** Interaction plot showing the effect of chemotherapy on proliferation in ER+ samples, measured the percentage of cells positive for Ki67. In red are samples with a relatively high proliferation rate before treatment (Ki67 ≥ 15), in orange are samples with a low pre-treatment proliferation rate (Ki67 < 15). **f** Kaplan–Meier curves of the samples shown in (**e**).

Unsupervised hierarchical clustering of the gene expression data revealed that the majority of post-treatment samples clustered together with the matched pre-treatment samples (Supplementary Fig. 6). Pathway enrichment analysis on differentially expressed genes between pre- and post-treatment samples, identified processes that were altered in multiple patients, including proliferation, cell cycle and DNA repair, cellular metabolism and extracellular matrix organization, and a strong enrichment of two stem-cell-associated gene signatures in post-treatment samples (Supplementary data file 3). ECM-related genes were upregulated in post-treatment samples while proliferation-related genes were downregulated, which supports our earlier observation of the role of these processes in chemotherapy response (Fig. 2a, b). Of note, ECM-related genes were upregulated in both ER+ and TN tumors after treatment (Fig. 2c), while the decrease in proliferation was most pronounced in ER+ tumors (Fig. 2d).

Although a high proliferation pre-treatment is related to pCR, a low proliferation rate after neoadjuvant treatment has been associated with a better prognosis, particularly in ER+ breast cancer where pCR is infrequent. We analyzed pre- and post-treatment Ki67 scores of 111 patients in our cohort, 94 of which were ER+/HER2−. All but one of the high-proliferative (Ki67 ≥ 35%) ER+ patients showed a sharp decrease in proliferation rate (Fig. 2e). Regardless of this decreased proliferation, pre-treatment high-proliferative ER+ tumors were still associated with a poor prognosis (Fig. 2f).

### Genomic differences between pre- and post-treatment samples may explain observed transcriptomic changes

**Fig. 3:**
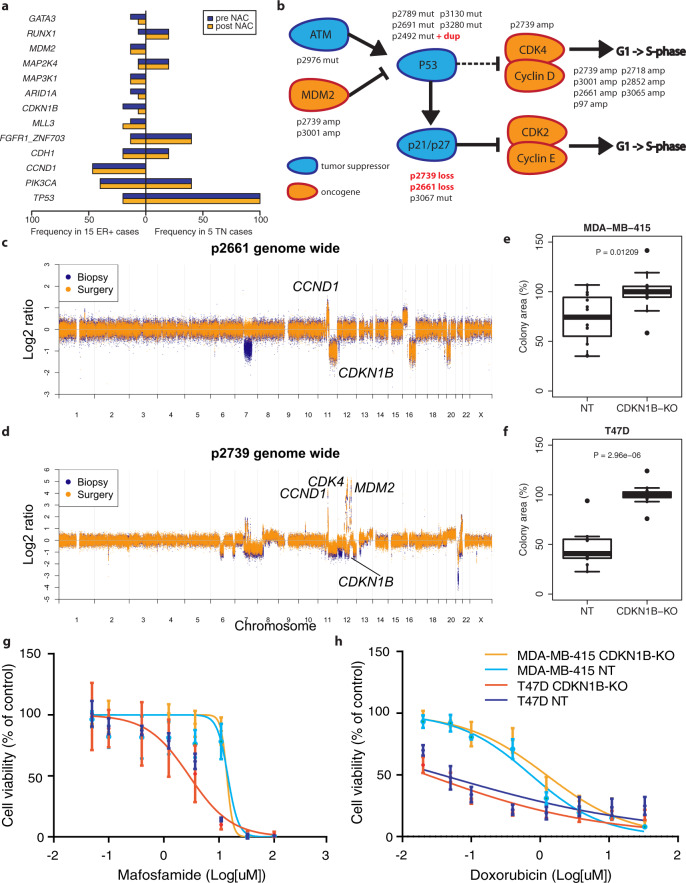
Genomic differences between breast tumors before and after chemotherapy. **a** Barplot of genes frequently aberrated in our cohort. Copy number changes and mutations are combined. In blue: frequency before treatment; orange: frequency after treatment. NAC neoadjuvant chemotherapy. **b** Simplified version of the cell cycle checkpoint pathway, highlighting genes aberrated in our cohort. Tumor suppressor genes are shown in blue, oncogenes in orange. Aberrated samples are indicated above/below the genes. Sample names in red indicate that the genetic aberration was not shared between the pre- and post-treatment sample. **c** Genome-wide copy number plot of patient s2661. Copy number changes of genes included in **b** are labeled. In blue, the copy number data of the pre-treatment sample; in orange, the post-treatment sample. **d** Genome-wide copy number plot of patient s2739. Layout is identical to (**c**). **e** Boxplots showing cell growth of MDA-MB-415 (*CCND1* amplified) breast cancer cell lines, with (left) or without (right) functional *CDKN1B*. **f** Boxplots showing cell growth of T47D (*CCND1* neutral) breast cancer cell lines, with (left) or without (right) functional *CDKN1B*. **g** Effect of *CDKN1B* knock-out on mafosfamide sensitivity in T47D (*CCND1* neutral) and MDA-MB-415 (*CCND1* amplified) breast cancer cell lines. Dark and lightblue: controls, red and orange: *CDKN1B* knock-out. **h** Effect of *CDKN1B* knock-out on doxorubicin sensitivity in T47D (*CCND1* neutral) and MDA-MB-415 (*CCND1* amplified) breast cancer cell lines. Dark and lightblue: controls, red and orange: *CDKN1B* knock-out. NT non-targeting control gRNA, KO knock-out with *CDKN1B* targeting gRNA. For box plots in this figure, the boxes indicate the interquartile range, while lower and upper bars correspond to the minimum and maximum non-outlier values of the data distribution. Outliers are defined as values outside 1.5× the interquartile range from the box. The center line indicates the median value.

Next, we analyzed the WES data to identify candidate genetic mutations and copy number alterations responsible for the observed transcriptomic changes (Supplementary data files 4 and 5). The most frequently aberrated genes in our dataset were *TP53* (5 TN and 3 ER+), *PIK3CA* (2 TN and 6 ER+) and *CCND1* (7 ER+) (Fig. 3a). Various mutations, copy number losses and amplifications were detected in the cell cycle checkpoint pathway (Fig. 3b). Most mutations in cancer driving genes (as defined by presence in the Cancer Gene Census 2021)^20^ were shared between pre- and post-treatment samples (Supplementary data file 4). However, we also observed some interesting exceptions: in one of the triple-negative tumors, we identified both a truncating *APC* and an activating β-catenin mutation in the post-treatment sample only. Both mutations lead to an increased activation of the Wnt-pathway^21^, which is in turn associated with a cancer stem cell phenotype^22^. Thus, these genetic alterations are in line with the strong enrichment of stem cell-like gene expression profiles we identified in the post-treatment samples.

In two different tumors, we detected copy number losses comprising *CDKN1B* in the pre-, but not in the matched post-treatment samples. Notably, both tumors also harbored a *CCND1* amplification (Fig. 3c, d). Since *CDKN1B* is a known tumor suppressor responsible for the control of cell cycle progression, we hypothesized that the loss of this gene would result in an increased proliferation rate and that restoration of this loss would reduce proliferation thus rendering the cells less sensitive to chemotherapeutic treatment. Potentially, the effect of *CDKN1B* loss is dependent on *CCND1* status, as *CCND1* is involved in the same pathway. We therefore tested the effect of *CDKN1B* knock-out in two cell lines: MDA-MB-415 (*CCND1* amplified) and T47D (*CCND1* neutral). Western blots confirmed the efficacy of the knock-out (Supplementary Fig. 7). In both cell lines, we observed a significant increase in cell growth in the *CDKN1B* knock-out cells compared to cells transfected with non-targeting gRNA (Mann–Whitney < 0.05, Fig. 3e, f). Thus, the restoration of *CDKN1B* copy number loss to a neutral stage in post-treatment samples can, by itself, be responsible for the decreased proliferation as observed in the post-treatment gene expression data of both tumors where we observed this restoration (Fig. 2d). We then investigated the effect of *CDKN1B* loss on chemotherapy response, as we observed that *CDKN1B* loss was only observed in the pre-treatment biopsies. This observation suggests that these pre-treatment samples would constitute a sensitive tumor subclone, which was eliminated in the post-treatment sample. We hypothesized that either the loss of *CDKN1B* alone, or the combination of *CDKN1B* loss and *CCND1* amplification would render cells more sensitive to therapy. To test this hypothesis, we knocked out *CDKN1B* in *CCND1* amplified (MDA-MB-415) and *CCND1* neutral (T47D) breast cancer cell lines. In neither cell line, *CDKN1B* knock-out resulted in a significant increase in therapy sensitivity (Fig. 3g, h), indicating that although *CDKN1B* knock-out resulted in increased proliferation, this does not translate in increased chemosensitivity. The relation between *CDKN1B* loss, decreased proliferation and chemotherapy resistance is more complex, with other—yet unknown—mechanisms at play.

### Association between *CCND1* amplification and therapy resistance

**Fig. 4:**
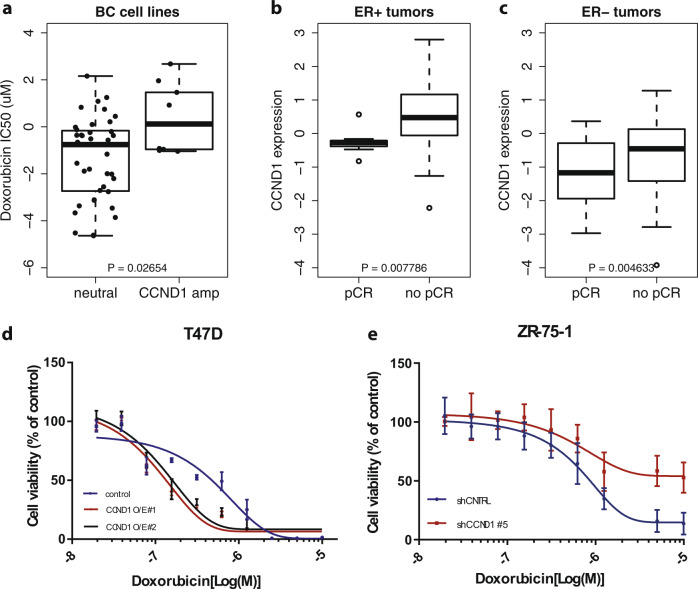
Exploration of the effect of *CCND1* amplification on chemotherapy sensitivity. **a** Boxplots showing sensitivity to doxorubicin in *CCND1* neutral (left) and amplified (right) breast cancer cell lines in the GDSC1000 database. **b** Boxplots showing the association between *CCND1* gene expression levels and response to neoadjuvant doxorubicin + cyclophosphamide in ER+ tumors from our neoadjuvant cohort. pCR pathological complete response. **c** Boxplots showing the association between *CCND1* gene expression levels and response to neoadjuvant doxorubicin + cyclophosphamide in triple negative tumors from our neoadjuvant cohort. pCR pathological complete response. **d** Effect of *CCND1* overexpression on doxorubicin sensitivity in T47D (*CCND1* neutral) breast cancer cell lines. Blue: control, red and black: *CCND1* overexpression. Mean ± SD values of two independent CCND1 overexpressing cell lines (O/E #1, O/E #2),with technical replicates are depicted (*n* = 3). **e** Effect of *CCND1* knock-down on doxorubicin sensitivity in ZR-75-1 (*CCND1* amplified) breast cancer cell lines. Blue: control, red: upon *CCND1* knock-down. Mean ± SD values of independent biological replicates are shown (*n* = 2). For box plots in this figure, the boxes indicate the interquartile range, while lower and upper bars correspond to the minimum and maximum non-outlier values of the data distribution. Outliers are defined as values outside 1.5× the interquartile range from the box. The center line indicates the median value.

In our search for genomic changes related to therapy resistance, we compared the frequencies of recurrent aberrations in the pre- and post treatment data to the frequencies of these events in an unselected population (BASIS cohort^23^) and TCGA^21^. *CCND1* was amplified in 44% of the ER+ cases, which is significantly higher than the reported frequencies in ER+ samples in the BASIS (20%, *p* = 0.05) and TCGA (19%, *p* = 0.02) cohorts. Since the selected pairs all represent samples that did not respond to treatment, we hypothesize that *CCND1* amplification may induce therapy resistance. To investigate this hypothesis, we first investigated the effect of *CCND1* copy number status and expression levels on response to doxorubicin in the GDSC1000 dataset^22^ and in our large neoadjuvant pre-treatment cohort (*n* = 317; described above). *CCND1* amplified breast cancer cell lines were more resistant to doxorubicin (Fig. 4a), and high *CCND1* expression levels were associated with non-response in both ER+ and ER− patient samples (Fig. 4b, c). To establish a potential cause-and-effect relationship between *CCND1* gene expression levels and treatment sensitivity, we altered gene expression levels in *CCND1* amplified (ZR-75) and *CCND1* neutral cell lines (T47D). The efficacy of the knock-down and overexpression experiments was confirmed using western blots (Supplementary Fig. 8). *CCND1* overexpression was not sufficient to induce doxorubicin resistance in *CCND1*-neutral breast cancer cell lines, nor did *CCND1* knock-down increase doxorubicin sensitivity in *CCDN1*-amplified cell lines, suggesting that *CCND1* is not the sole driver of doxorubicine resistance in this setting. Importantly, both cell lines models showed the opposite phenotype of what was expected (Fig. 4d, e), which could potentially be explained by the cell cycle effects of Cyclin 1, known to impact response to chemotherapeutics^24^.

Summarizing, we could confirm the association of *CCND1* levels with doxorubicin response in the GDSC1000 cell line data and our large neoadjuvant cohort. However, we were not able to model the effect of *CCND1* expression on chemotherapy sensitivity in cell lines as a single driver event, with or without *CDKN1B* loss, illustrating that other biological mechanisms are also involved.

## Discussion

Here we showed, based on molecular profiling of 317 pre-NAC breast cancer biopsies, that gene expression levels of proliferation, immune response, and extracellular matrix organization, jointly predict pathological complete response to chemotherapy in both TN and ER+ breast cancer. Gene expression profiling of matched pre- and post-treatment samples of 22 chemotherapy-resistant tumors revealed a decrease in proliferation and a strong enrichment in stem cell-related signatures in post-treatment samples, which was most prominent in ER+ tumors. In addition, WES analysis implicated *CCND1* amplifications in chemotherapy resistance.

In most ER+ tumors, we observed a mild to strong decrease in proliferation after chemotherapy, analogous to earlier studies^25^. It has been shown that a low tumor cell proliferation after treatment is a prognostic factor, specifically in ER+ breast cancer^26^, but we could not validate this observation in our dataset. In fact, of the 13 recurrences in our dataset, 6 showed a post-treatment Ki67 score of ≤5%. We therefore hypothesize that some highly proliferative ER+ tumor cells enter a dormant- or senescent-like state under duress of chemotherapy, but re-enter the cell cycle once the therapy is removed. DNA damage-induced senescence and senescence reversal in solid tumors have been studied extensively (as reviewed in ref. ^27^). Tumor cells that escape, or reverse, senescence may be more resistant to drugs and associated with a poor prognosis^28^. Future studies should establish whether or not senescence is indeed a key player in chemotherapy resistance in ER+ breast cancer.

Proliferation also plays a role in predicting response to chemotherapy: high proliferation levels of treatment-naïve samples have been associated with an increased chance of response to chemotherapy in various studies^29,30^. We could confirm this association in our dataset of patients receiving neoadjuvant chemotherapy as well. Additionally, we found associations between chemotherapy response and genes involved in immune response and extracellular matrix organization (ECM). These associations have also been identified before, either through pathology review/immunohistochemistry^5,31^ or gene expression profiling^7,15,29,32,33^. Interestingly, Park et al. show that chemotherapy induces dynamic changes in the immune microenvironment, that are different between TN and ER+ tumors, where TN are the most immunogenic, and could be primed by NAC for responding to immuno-oncology therapies^33^. In contrast to Park et al. we did not identify immune related gene sets as one of the key pathways between pre and post-treatment samples, which might be due to enrichment of luminal-type cancers in our pre–post chemotherapy sample set. We observed that proliferation and ECM both appear to be predictive of chemotherapy response in treatment-naïve samples, and to change under duress of chemotherapy treatment. Kim et al. show up to the single cell level that ECM degradation is upregulated in chemoresistant tumor cells after NAC^32^. Although the involvement of these pathways in chemotherapy response have been shown before, we substantiate these findings in a large samples series of both TN and ER+ treatment naïve biopsies.

Our WES data revealed associations between well-known cancer genes and chemotherapy response, such as *CTNNB1* and *APC*, *TP53, CDKN1B* and *CCND1*. In one triple negative post treatment sample both an activating *CTNNB1* and a truncating *APC* mutation were observed. These two mutations cause dissociation of β-catenin from APC, leading to accumulation of β-catenin in the nucleus and increased transcription of its target genes, including *CCND1*^34,35^. Unfortunately, gene expression data was not available for this patients’ post-treatment sample to test the hypothesis of increased expression of those target genes. Mutations in *APC* and *CTNNB1* are quite common in several tumor types, including colorectal^36^, endometrial^37^, and hepatocellular carcinoma^38^, but very rare in breast cancer, although deregulation of the Wnt/𝛽-catenin pathway is relatively common^39^. Notably, the disease in this patient progressed very rapidly even under treatment, and the patient passed away within 6 months after the start of treatment indicating a highly aggressive tumor. Interestingly, Brastianos et al. identified an activating *CTNNB1* mutation in a brain metastasis of a HER2+ breast cancer patient, which was also not detectable in the primary tumor or the other two brain metastasis samples^40^. Various Wnt inhibitors are currently under investigation for the treatment of different solid tumors (e.g. clinical trials NCT01351103, NCT02521844, and NCT02675946). If these inhibitors show potential and get approval, they may be extremely valuable for a small subset of breast cancer patients.

We show that *CCND1* amplification or overexpression is associated with chemotherapy resistance in both ER+ and TN breast tumors: we observed a significant enrichment of *CCND1*-amplified tumors in our cohort of chemotherapy resistant samples, *CCND1* amplification was associated with higher doxorubicin IC_50_s in breast cancer cell lines, and expression of *CCND1* was significantly higher in tumors that did not achieve a pCR in our validation cohort. In a similar study by Balko et al. *CCND1* amplifications were also significantly enriched in chemotherapy-resistant TN tumors, together with amplifications of other Cyclin D-genes and *CDK6*^41^. *CCND1* amplification is one of the most common drivers of (ER+) breast cancer, present in ~20% of cases^23^, and it is associated with a poor prognosis^42^. CDK4/6 inhibitors, targeting the direct binding partners of *CCND1*, show promise for ER+ breast cancer. However, its method of action does not appear to be related to *CCND1* amplification status. The PALOMA-1 trial, randomizing ER+/HER2− patients between hormone treatment + palbociclib or hormone treatment only, initially investigated the added effect of CDK-inhibition in two separate cohorts. One cohort included patients with an expected benefit of the drug, because of *CCND1* amplification or loss of *CDKN2A*, while the second cohort included all other patients. Even though a significant survival benefit was observed in both cohorts, the effect was much larger in the unselected group than in the group with *CCND1* amplifications^43^. With our in vitro studies, we could not reconstitute the phenotype seen in our clinical data, i.e. overexpression of *CCND1* did not result in treatment resistence, nor did *CCND1* knockdown result in sensitivity. Interestingly, similar studies into tamoxifen resistance, also showed that 11q13 alterations are associated with drug resistance, however cyclin D1 was neither here the sole driver of resistance^44,45^.

This study has some strengths and limitations. A major strength is that both gene expression analysis of a large cohort of treatment naïve tumor samples has been performed, as well as a deep genomic characterization of pre- and post-treatment samples. Consequently, we could both study pre-treatment factors as well as resistance mechanisms, and validate them over the two datasets. Indeed, we could validate that high CCND1 amplification confers treatment resistance with gene expression data of our large treatment naïve cohort, showing an association to non-response. Another strength is the focus on two breast cancer subtypes. While many studies focus on triple-negative breast cancer, we here see similar processes in luminal-type breast cancer. A third strength is the deep whole exome characterization of the pre-and post-treatment samples, on tumor-enriched (minimum tumor percentage or 50%) samples. This study has also some limitations. First, the number of pre–post treatment samples is small. It is difficult to obtain post-treatment samples, as tumor percentage is quite low, and tissue has often been damaged by the neoadjuvant chemotherapy. However, we found common mutations, and were able to validate some markers in vitro and in clinical datasets, like CCND1 and CDKN1B. Second, we only performed bulk sequencing. Therefore, we could not precisely characterized mechanisms of gain or loss between pre-and post-treatment samples. For example, we found a CTNNB1 mutation in a post treatment sample. We cannot exclude the fact that this mutation was already present pre-treatment, and was selected for in the post-treatment sample.

In conclusion, this study shows that comparing pre- and post-treatment samples has the potential to reveal mechanisms of therapy resistance. Our results show a wide range of distinct, but related mechanisms, with a prominent role for proliferation-related genes and *CCND1* in particular.

## Methods

### Patient cohort and sample selection

Biopsies of primary breast tumors were collected and snap-frozen in liquid nitrogen prior to treatment from women with locally advanced breast cancer at the Netherlands Cancer Institute between 2000 and 2013. All patients had received neoadjuvant treatment as part of ongoing clinical trials (NCT00448266, NCT01057069), or were treated off protocol according to the standard arms of one of these studies. Patient characteristics and treatment strategies are listed in Tables 1 and 2. For the analysis of matched samples before and after treatment, we used the following additional selection criteria: (1) no pCR after receiving standard of care neoadjuvant chemotherapy; (2) >50% tumor cells in all samples; (3) availability of fresh frozen material of all samples; and (4) availability of matched blood. The ethical committee of the Netherlands Cancer Institute approved the studies and all patients gave informed consent.

### Pathology

All tissue sections were reviewed by a consultant breast cancer pathologist (J.W.). Samples were scored as positive for ER and/or PR by immunohistochemistry (IHC), when at least 10% of the tumor cells nuclei showed staining of the ER or PR, respectively. A sample was scored as being HER2 positive when either a strong membrane staining (3+) could be observed by IHC or if CISH revealed amplification of HER2 in samples with moderate (2+) membrane staining at IHC. Ki67 staining was performed with the MIB1 antibody (Dako, Glostrup, Denmark), dilution 1:250. Chemotherapy response was assessed by microscopic examination of the surgery resection specimen. We both used the neoadjuvant response index (NRI)^8^ as well as pCR to assess response. The complete absence of any invasive tumor cells in both the breast and the lymph nodes was considered as a pCR.

### Exome seq library prep and data processing

Isolation of DNA from fresh frozen specimen was performed with a DNA mini kit (Qiagen, Venlo, the Netherlands). Matched normal DNA was obtained from peripheral blood and extracted by DNAzol and purified with Qiagen DNeasy kit. DNA libraries were constructed using the KAPA LTP Library Preparation Kit (KK8234), enriched for exome sequences using the Agilent SureSelect XT2 Human Exome Target Enrichment system (5190–8872), quantified on a DNA7500 assay chip (5067–1506) on an Agilent 2100 Bioanalyzer and subsequently sequenced 100 basepair paired-end on a HiSeq 2500 System of Illumina (see Supplemental methods for further details).

Reads were aligned to the human reference genome version 37.75 (hg19) using bwa v0.7.17^46^. Duplicate removal, base recalibration, variant calling and annotation was done using the GATK Haplotype caller v3.4^47^, SNPEff v4.1^48^ and SNPSift v4.1^49^. Somatic variants were identified using Strelka v2.9.10^50^, annotated using the Ensembl Variant Effect Predictor v83^51^ and genotyped per patient in exome sequencing and RNAseq data using samtools v1.10 pileup^52^ and custom perl scripts. Only somatic variants with an allele frequency of >10% in either pre- or post-treament sample and an effect on the protein coding sequence were retained for further analyses. Copy number estimations were generated using CNVkit v0.9.7^53^, using all blood samples as a common reference. Copy number log ratios and somatic allele frequencies were adjusted for estimated tumor percentage (see Supplemental Methods for details). Following this adjustment, copy number changes between matched pre- and post-treatment samples were determined by subtracting the log ratio per probe of the biopsy from the surgery specimen, and segmenting the obtained values.

### Microarray analyses, RNA-seq library prep and data processing

The microarray data was generated and analyzed^54^ and made available through the GEO database, accession GSE34138. Briefly, samples were hybridized to Illumina WG6 v3 microarray chips. Genes that were not detected above background level in at least one sample were excluded from the analysis. The data were normalized by applying between array simple scaling and a subsequent log2 transformation.

RNA was isolated from samples with a tumor percentage >50%, from thirty 30-μm cryosections. A 5-μm section halfway through the biopsy was stained for hematoxylin and eosin and analyzed by a pathologist for tumor percentage. Total RNA was isolated with RNA-Bee (Bio-Connect, Huissen, The Netherlands, Cat No. CS-100B), DNase-treated by using the Qiagen RNase-free DNase Set (Qiagen, Venlo, The Netherlands, Cat No. 79254) and RNeasy spin columns (Qiagen, Cat No. 74104) and dissolved in RNase-free H_2_O.

Quality and quantity of the total RNA was assessed by the 2100 Bioanalyzer using a Nano chip (Agilent, Santa Clara, CA); samples having RIN > 6.4 were subjected to library generation. mRNA libraries were generated using the TruSeq RNA Library Preparation Kit v2 (Illumina Inc., San Diego, Cat. No. RS-122-2001/2) according to the manufacturer’s instruction (Part # 15026495 Rev. B). The libraries were analyzed on a 2100 Bioanalyzer using a 7500 chip (Agilent, Santa Clara, CA, USA), diluted and pooled equimolar into a 10 nM sequencing pool containing 9 libraries each, and sequenced with 50 basepair single reads (pre-treatment samples only) or 65 basepair single reads (pre–post treatment set) on a HiSeq2000 using V3 chemistry (Illumina Inc., San Diego).

Reads were aligned to the human transcriptome (Homo_sapiens.GRCh37.75.gtf) using Tophat v2.1^55^. Readcounts per gene were calculated using lcount^56^, and normalized using DESeq2 v1.22.0^57^. The SVA R package v3.30.1^58^ was used to combine and batch correct gene expression datasets. Gene expression levels were associated with chemotherapy response using samr v3.0;^59^ differential gene expression between pre- and post-treatment samples was analyzed using DESeq2 v1.22.0^57^. Subsequent pathway/GO-term enrichment was performed using the Reactome Cytoscape plugin v3.1.0^60,61^ and geneset enrichment was performed using GSEA v4.1.0^62^.

The most significant genes from the samr analysis (FDR < 5%) were selected for a ‘core enrichment’ set, based on unsupervised clustering and pathway and GO-term enrichment (Supplementary data file 1). Metagene scores for all three processes were created by calculating the median expression of genes assigned to each cluster. Expression above or below the metagene median was used to stratify patients into a HHL (proliferation high, immune high, ECM low) or LLH (proliferation low, immune low, ECM high) profile. Statistical analysis of recurrence-free survival in these profiles was done using Kaplan–Meier estimates.

RNA-seq reads of the pre–post dataset were also aligned to the human reference genome version 37.75 (hg19) using bwa 0.7.17^46^ to be able to confirm somatic mutations and indels in the expression data.

### *CDKN1B* and *CCND1* knockout/knockdown and overexpression experiments

The LentiCRISPRv2 vector was a kind gift from Feng Zhang (Addgene, plasmid no. 52961). sgRNA sequences for *CDKN1B* knock-out^63^ and negative control^64^ are listed in the Supplemental Methods. Cloning of the sgRNAs was performed and vectors were validated by Sanger sequencing^63^. We produced concentrated lentiviral stocks, pseudotyped with the VSV-G envelope, by transient cotransfection of four plasmids in 293T cells^65^. Viral titers were determined using the quantitative PCR (qPCR) lentivirus titration kit from Abm (LV900).

Culture conditions for 293T, T47D and MDA-MB-415 are listed in the Supplemental methods. Transductions were performed by adding diluted viral supernatant to the cells in the presence of 8 μg/mL polybrene (Sigma). Cells were transduced at a multiplicity of infection (MOI) of 10 for 24 h, after which the medium was refreshed. Harvesting of cells for genomic DNA Genomic DNA from cell pellets was isolated using the Gentra Puregene genomic DNA isolation kit from Qiagen. PCR amplification of Cdkn1b exon 1 was performed with primers spanning the target site (Supplemental methods) and 1 μg of DNA template using the Q5 high-fidelity PCR kit from New England Biolabs. Amplicons were run on 1% agarose gel, and gel purification was performed using the Isolate II PCR and gel kit from Bioline. PCR products were Sanger-sequenced using the FW primer, and CRISPR/Cas9-induced editing efficacy was quantified with the TIDE algorithm (http://tide.nki.nl)^66^. Non-transduced cells were used as a negative control in all genomic DNA amplifications, and only TIDE outputs with *R*^2^ > 0.9 were considered.

Three short hairpin RNAs targeting *CCND1* were selected from the TRC Human v2.0 library (#1: TRCN0000295876; #3: TRCN0000288598; #5: TRCN0000295874). Non-targeting short hairpin was used as a negative control. Lentivirus were produced in 293T cells. Supernatant was collected and transferred to ZR-75-1 cells together with polybrene (8 μg/mL). Puromycin was added 48 h after infection for selection. Lentiviral plasmids containing the *CCND1* open reading frame were obtained from CCSB-Broad Lentiviral Expression library (#1: #101926645) and from the Jonkers laboratory at the Netherlands Cancer Institute (#2: Lenti737-ccnd1-P2A-CRE). Medium was harvested, polybrene was added at a final concentration of 8 μg/μL and it was transferred to T47D cells for virus infection. Medium was refreshed 24 h after infection.

### Cytotoxicity and cell viability experiments

T47D-CDKN1BKO and MDA-MB-415-CDKN1BKO cells were seeded on 12-well plates for mafosfamide and doxorubicin cytotoxicity assays at densities of 2000 cells/well (T47D) or 2500 cells/well (MDA-MB-415). One day after seeding, ranges of concentrations of mafosfamide (Niomech, 0–100 μM) or doxorubicin (Sigma, 0–33 μM) were added to the cells in triplicate. After 3 days of drug treatment, cell viability was measured with CellTiter-Blue (Promega) using a 96-well plate reader (Tecan). Cells were then washed with PBS, fixed with 4% paraformaldehyde and stained with 0.1% crystal violet. Plates were imaged and cell densities were measured using Image J Colony Area plugin.

For shCCND1-ZR-75-1 cells and T47D cells overexpressing CCND1, cells were seeded at a density of 500 cells/well in a 384-well plate. 24 h after seeding, cells were treated with ranging concentrations (0–10 μM) of doxorubicin (MedChemExpress: HY-15142) for a period of 7 days and cell viability was measured using Cell-Titer Glow (Promega).

### Reporting summary

Further information on research design is available in the Nature Research Reporting Summary linked to this article.

## Supplementary information

Supplemental material

Supplemental data set 1

Supplemental data set 2

Supplemental data set 3

Supplemental data set 4

Supplemental data set 5

Reporting Summary Checklist

## Data Availability

The WES data is available through EGA: EGAS 0000100587 (dataset EGAD00001008442). The microarray RNA data is available through GEO: GSE34138. The RNAseq data is available through EGA (raw data): EGAS 00001005876 (EGAD00001008421 and EGAD00001008433) and GEO (processed data): GSE191127 and GSE192341.

## Code availability

The code used in this study can be accessed at: https://github.com/NKI-CCB/prepost_neoadjuvant.
