# Supplementary material for: Comprehensive characterization of pre- and post-treatment samples of breast cancer reveal potential mechanisms of chemotherapy resistance
Source: NPJ Breast Cancer. 2022 May 6;8:60. doi: 10.1038/s41523-022-00428-8 (PMC9076915; doi:10.1038/s41523-022-00428-8)

Supplementary information

Supplementary Methods

Exome sequencing

We started with 500-1000 ng sheared DNA input material for the construction of DNA libraries for Illumina sequencing using the KAPA LTP Library Preparartion Kit (KK8234), according to manufacturer's protocol. A pool of 8 DNA libraries was enriched for exome sequences using the Agilent SureSelect XT2 Human Exome Target Enrichment system (5190-8872). We used 1:1 diluted exome capture library, all other went according to manufacturer's protocol (G9630-90000). The enriched DNA libraries were quantified on a DNA7500 assay chip (5067-1506) on an Agilent 2100 Bioanalyzer system before sequencing 100 basepairs paired-end on a HiSeq 2500 System of Illumina. For tumor samples, pools of 8 libraries were sequenced over 4 lanes, while libraries of matched normal tissue were sequenced over 2 lanes.

To estimate these tumor cell percentages, we genotyped SNPs from the Genome of the Netherlands project (23) with an allele frequency of > 0.25 (n=3019964) in all tumor samples using samtools mpileup(24) and custom perl scripts. Tumor cell percentage was estimated by determining the average major allele frequency at genomic deletions as determined by cnvkit (log ratio < -0.2). These estimated percentages were used to adjust the somatic variant allele frequencies and the copy number log ratios.

Validation of the metagene analysis in TCGA samples

Breast cancer samples from the TCGA Pan-Cancer Atlas [19] were downloaded from CBioPortal (https://www.cbioportal.org/study/clinicalData?id=brca_tcga_pan_can_atlas_2018). PFS was used to determine clinical outcome, as recommended [19]. Since ER/PR/HER2-status were not available, we selected samples with the basal subtype as a proxy for TN samples, and samples with the Luminal A/B subtype as luminal samples. Gene expression levels were taken from the Z-transformed batch corrected RSEM data. Based on gene name identifiers, 280 out of the 299 core enrichment genes were present in this dataset. With this minor difference, calculation of the three metagene scores, stratification of samples into HHL, LLH and 'other' profiles and statistical analysis of survival were performed as described above for the neoadjuvant dataset.

Western blot analysis

In the *CDKN1B* knock-out experiments, T47D and MDA-MB-415 cells were incubated with lysis buffer containing protease inhibitors (Complete, Roche) for 20 minutes on ice. After centrifugation, protein concentrations were determined using the BCA assay (Pierce) and 50 μg protein of each lysate was loaded on a 4-12% Bis-Tris acrylamide gel for gel electrophoresis in MOPS buffer (Invitrogen). After semi-dry blotting on a nitrocellulose membrane (Bio-Rad), western blots were performed. Membranes were blocked with 5% non-fat dry milk in TBS-T (10 mM Tris-HCl pH 7.5, 150 mM NaCl, 0.05% Tween-20) and subsequently incubated with rabbit anti-p27 Kip1 (Cell Signaling 2552, 1:1000) or rabbit anti-Cyclin D1 (Abcam 16663, 1:200) in TBS-T+ (TBS-T with 1% non-fat dry milk). Beta-catenin was taken along as a loading control (Cell signaling #8480, 1:2000). After washing with TBS-T+, membranes were incubated with goat anti-rabbit HRP labeled secondary antibodies (Dako, 1:2000) in TBS-T+ and washed with in TBS-T. Signal was detected on film using ECL (Pierce).

In the *CCND1* O/E and knock-down experiments, cells were lysed and proteins were collected with 2xLaemmli buffer (120 mMTris, 20% glycerol, 4% SDS). Amount of protein was determined by using BCA assay (23227, Thermo Fisher Scientific) as described by manufacturer's instructions. Equal amounts of protein were run on SDS-PAGE and subsequently transferred to a nitrocellulose membrane. Western blot analyses were performed by blocking the membranes with 3% BSA-PBS-T (137mM NaCl, 10mM Na2HPO4, 1.5mM KH2POA4, 2.6mM KCl, 0.1% Tween-20), washed with 1x PBS-T and incubated with 3%BSA-PBS-T containing antibodies against CCND1 (ab239794, 1:1000) and Hsp90 (sc-7947,1:1000). Blots were washed with 1xPBS-T, incubated with secondary antibody, and imaged using Odyssey Infrared Imaging System. All blots were processed in parallel and derived from the same experiment.

***CDKN1B* knock-out**

The sgRNA targeting human Cdkn1b exon 1 (GGGTTAGCGGAGCAATGCGC) was selected from the GeCKO version 2 human gRNA library (34). A non-targeting sgRNA (TGATTGGGGGTCGTTCGCCA) was used as negative control (35). PCR primers for CDKN1B exon 1 amplification were: FW_Cdkn1b, GGCAAGTACGAGTGGCAAGA; and RV_Cdkn1b, AGCACTGAACACCTAAGACCA.

293T cells for lentiviral production were cultured in Iscove's medium (Life Technologies) containing 10% FBS, 100 IU/mL penicillin, and 100 µg/mL streptomycin. T47D (CCND1 normal) and MDA-MB-415 (CCND1 amplified) cells were cultured in RPMI 1640 medium (Gibco) containing 10% FBS, 100 IU/mL penicillin, 100 µg/mL streptomycin and 10 µg/ml insulin.

Supplementary Data files

Supplementary data file 1: **Genes significantly associated with NRI in the neoadjuvant cohort, with pathway annotations.** Subsequently the genes correlated with NRI (red positive correlation with NRI; green negative correlation with NRI) are shown; the down regulated GO-terms, the down regulated pathways; the up regulated GO-terms, the up-regulated pathways; and the upregulated CIBERSORT terms.

Supplementary data file 2: **Sequencing statistics of the pre-post tumor pairs.** The sequencing statistics for exome sequencing and RNA sequencing are shown for all pre-post tumor pairs.

Supplementary data file 3: **Differentially expressed genes between pre- and post-chemotherapy samples, with pathway annotations.** Subsequently all significant differentially expressed genes between pre-and post-samples are shown; the GSEA hallmarks of these genes; gene sets from the molecular signature database CGP; and processes of the Reactome database.

Supplementary data file 4: **Variants in pre- and post-chemotherapy samples.** All somatic variants in the paired pre-and post-treatment samples are shown here.

Supplementary data file 5: **Copy number alterations in pre- and post-treatment samples.** All copy number changes in the paired pre-and post-treatment samples are shown here.

Supplementary figures

Supplementary Figure 1: Kaplan-Meier survival plot of recurrence free survival (RFS) of all triple-negative patients split for pCR status.

Supplementary Figure 2: Kaplan-Meier survival plot of recurrence free survival (RFS) of all ER+ patients split for pCR status.

Supplementary Figure 3: Correlation of Ki67 IHC and proliferation-associated genes.

Supplementary Figure 4: Kaplan-Meier survival plots for TCGA breast cancer basal- type subset, stratified by HHL and LLH gene profiles. A. Full basal TCGA cohort. B. High risk group (N+ or T3/4 group). C. Lymph node negative subgroup. D. Lymph node positive subgroup. E. Low stage (T1/T2) subgroup. F. High stage subgroup (T3/4). P-values by log-rank test are shown.

Supplementary Figure 5: Kaplan-Meier survival plots for TCGA breast cancer luminal- type subset, stratified by HHL and LLH gene profiles. A. Full luminal-type TCGA cohort. B. High risk group (N+ or T3/4 group). C. Lymph node negative subgroup. D. Lymph node positive subgroup. E. Low stage (T1/T2) subgroup. F. High stage subgroup (T3/4). P-values by log-rank test are shown.

Supplementary Figure 6: Hierarchical unsupervised clustering of gene expression data of pre- and post-chemotherapy samples

Supplementary Figure 7: Western blot analysis p27 and cyclinD1

Supplementary Figure 8: Western blot analysis for therapy resistance experiments. Mean ± SD values of independent biological replicates are depicted (n=2).

Suppl Fig 1

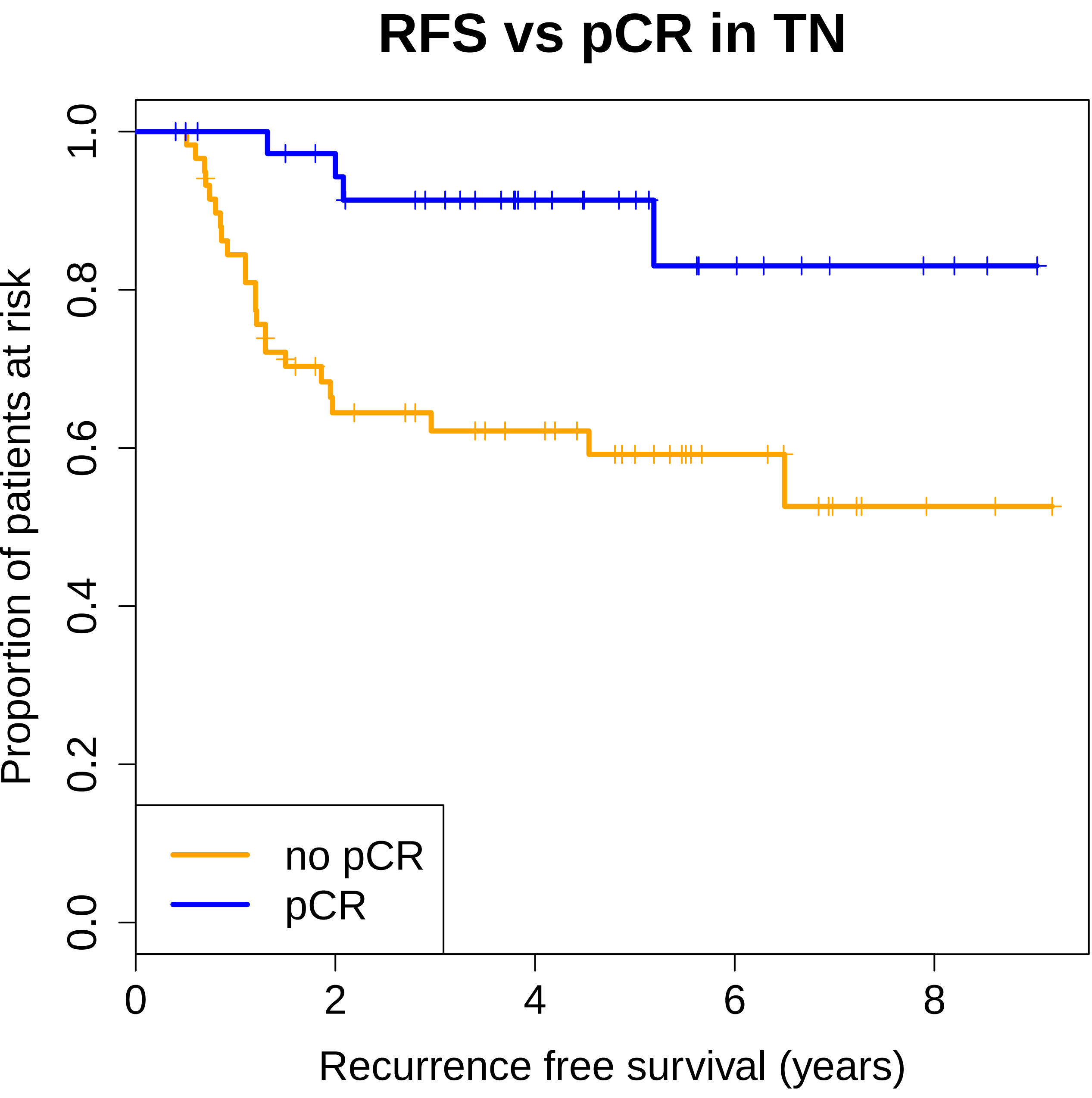
RFS vs pCR in TN
Proportion of patients at risk
1.0
0.8
0.6
0.4
0.2
0.0
0
2
4
6
8
Recurrence free survival (years)
no pCR
pCR

Suppl Fig 2

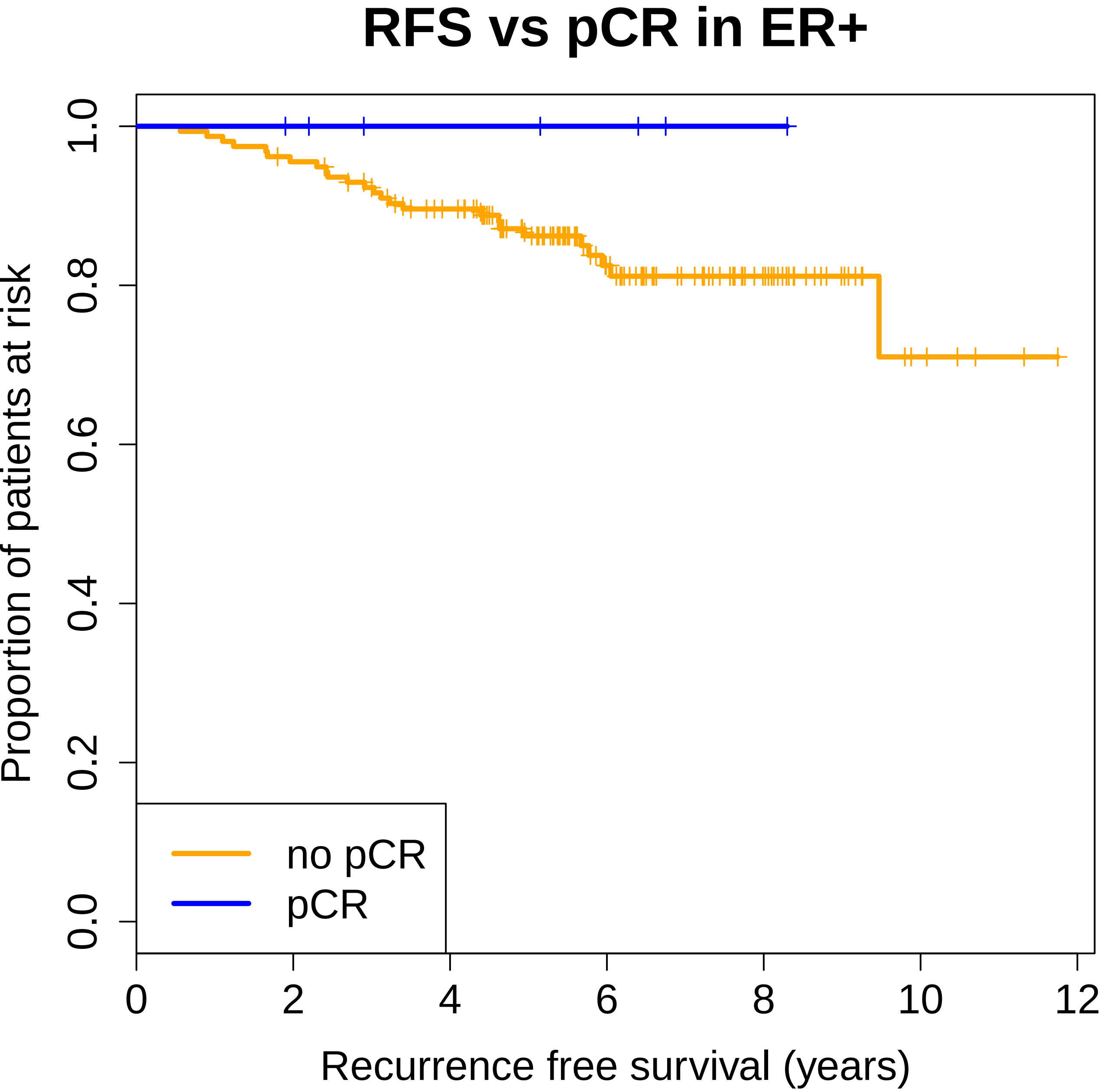
RFS vs pCR in ER+
Proportion of patients at risk
1.0
0.8
0.6
0.4
0.2
0.0
0
2
4
6
8
10
12
Recurrence free survival (years)
no pCR
pCR

Suppl Fig 3

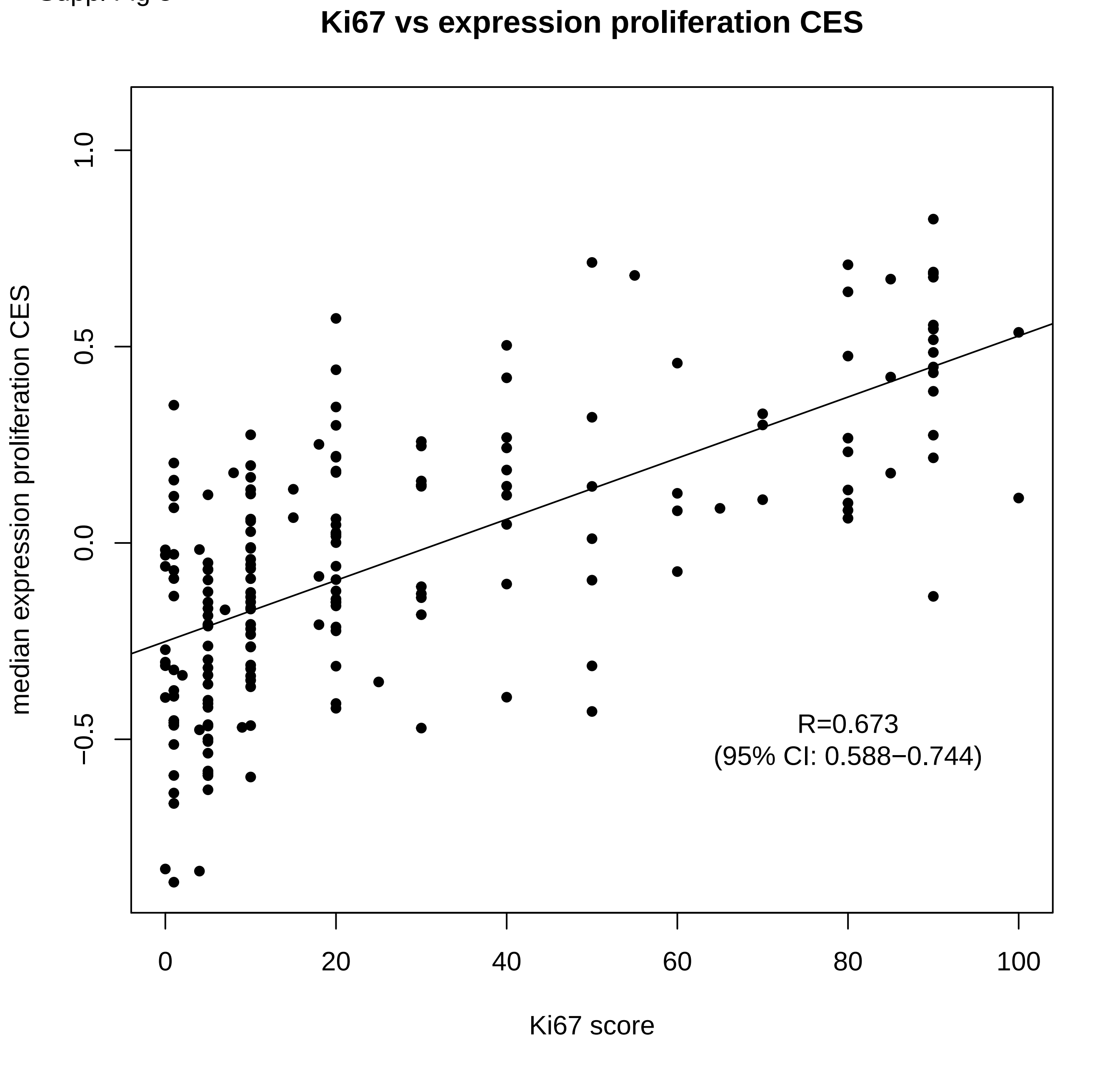
Ki67 vs expression proliferation CES
median expression proliferation CES
Ki67 score
R=0.673
(95% CI: 0.588−0.744)
1.0
0.5
0.0
−0.5
0
20
40
60
80
100

Suppl Fig 4

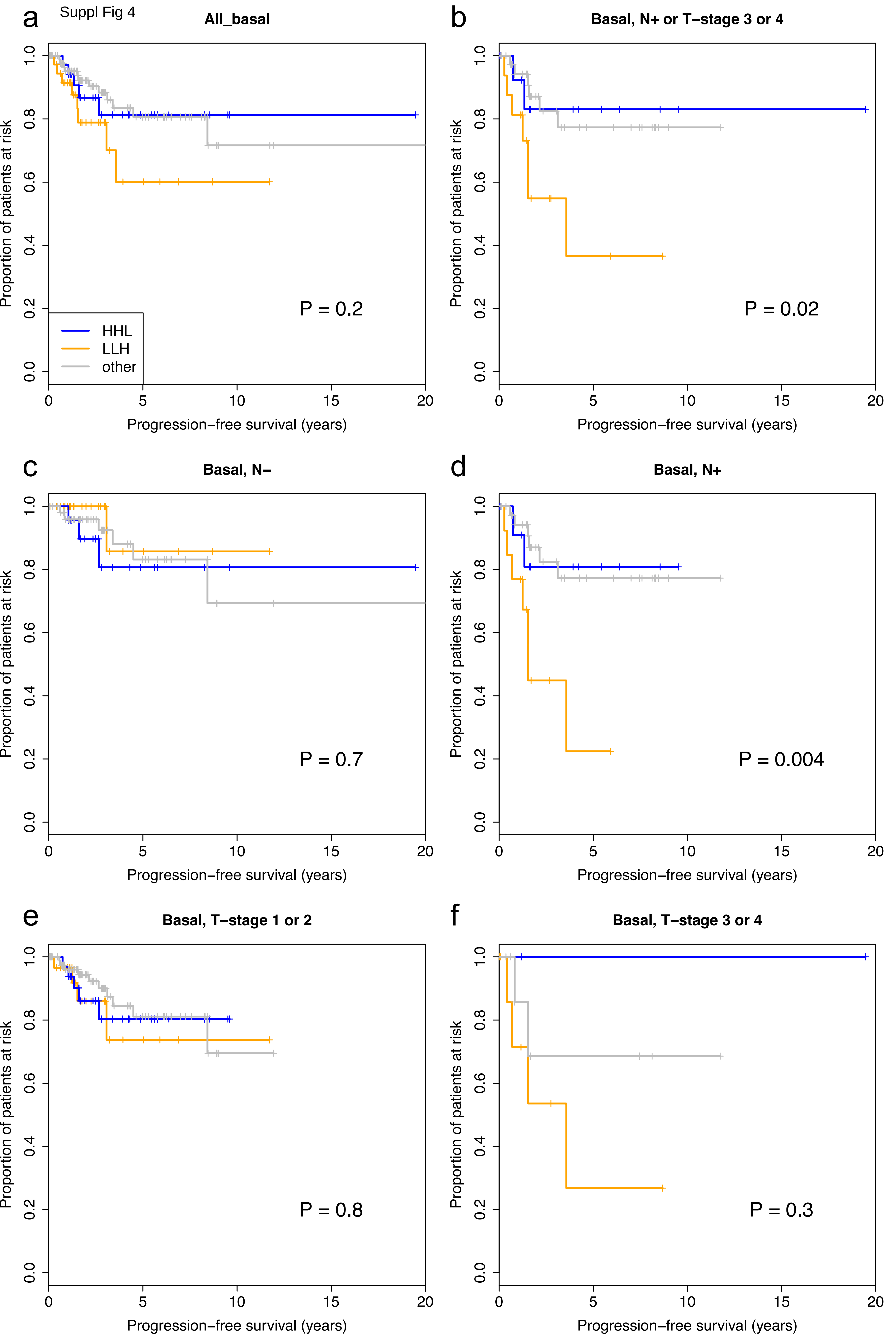

a
All_basal
Proportion of patients at risk
Progression−free survival (years)
P = 0.2
HHL
LLH
other
b
Basal, N+ or T−stage 3 or 4
P = 0.02
c
Basal, N−
P = 0.7
d
Basal, N+
P = 0.004
e
Basal, T−stage 1 or 2
P = 0.8
f
Basal, T−stage 3 or 4
P = 0.3

Suppl Fig 5

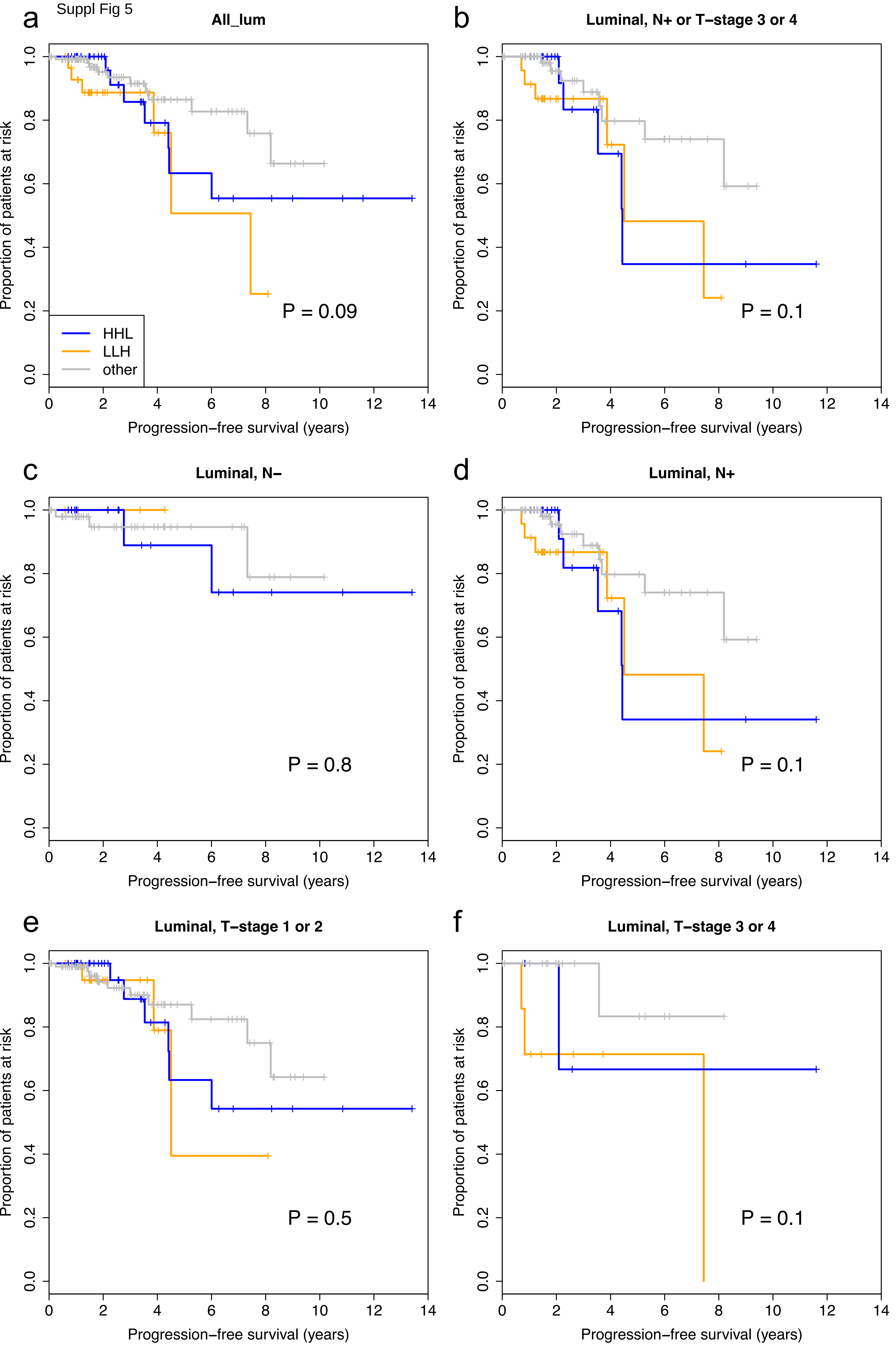

a
All_lum
P = 0.09
HHL
LLH
other
b
Luminal, N+ or T−stage 3 or 4
P = 0.1
c
Luminal, N−
P = 0.8
d
Luminal, N+
P = 0.1
e
Luminal, T−stage 1 or 2
P = 0.5
f
Luminal, T−stage 3 or 4
P = 0.1
Proportion of patients at risk
Progression−free survival (years)
0.0
0.2
0.4
0.6
0.8
1.0
0
2
4
6
8
10
12
14

Suppl Fig 6

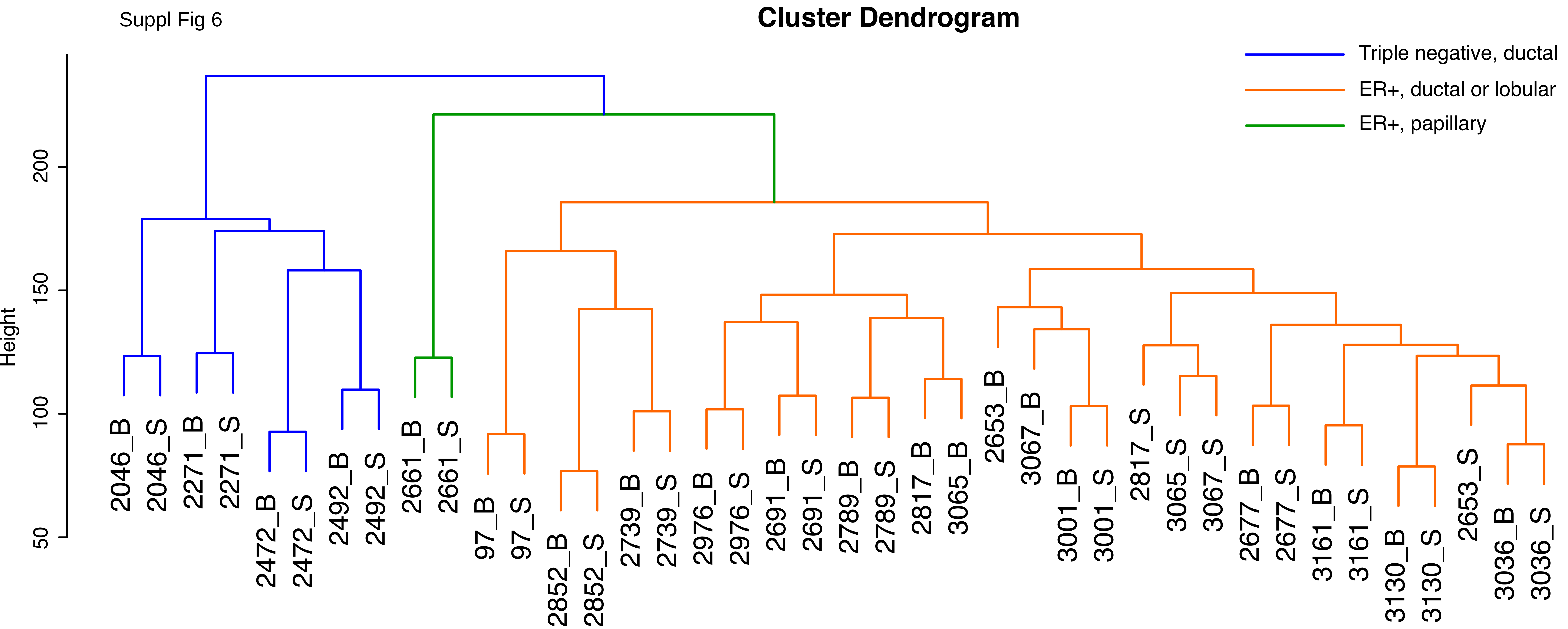

Cluster Dendrogram
Triple negative, ductal
ER+, ductal or lobular
ER+, papillary
Height
50
100
150
200
2046_B
2046_S
2271_B
2271_S
2472_B
2472_S
2492_B
2492_S
2661_B
2661_S
97_B
97_S
2852_B
2852_S
2739_B
2739_S
2976_B
2976_S
2691_B
2691_S
2789_B
2789_S
2817_B
3065_B
2653_B
3067_B
3001_B
3001_S
2817_S
3065_S
3067_S
2677_B
2677_S
3161_B
3161_S
3130_B
3130_S
2653_S
3036_B
3036_S

Suppl Fig 7

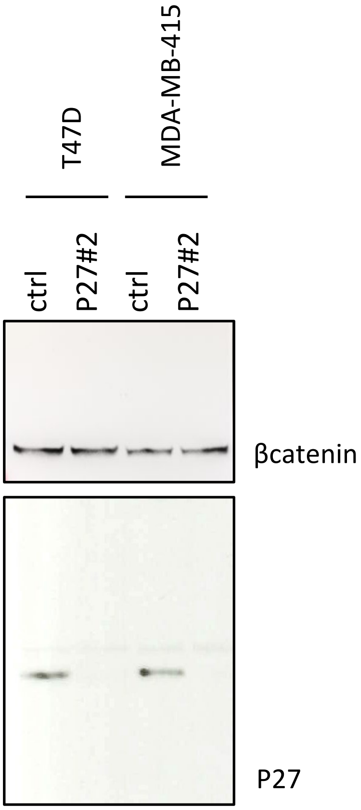

βcatenin/ 92kD
Rb-anti-βcatenin
Cell signalling #8480
1:2000

P27 / 27kD
Rb-anti-p27kip
Cell signalling #2552
1:1000

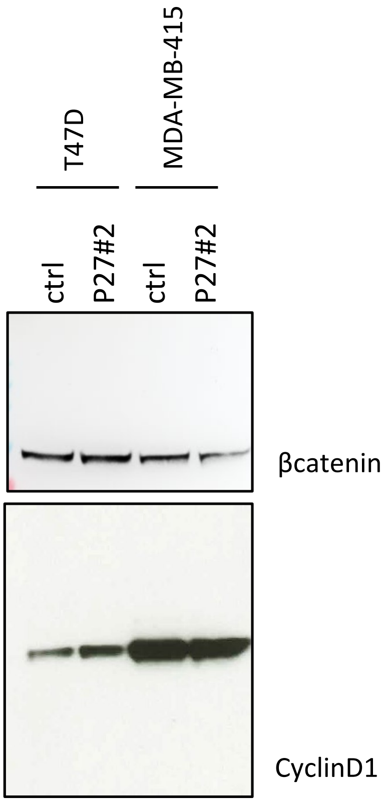

βcatenin/ 92kD
Rb-anti-βcatenin
Cell signalling #8480
1:2000

CyclinD1 / 33kD
Rb-anti-cyclinD1 (SP4)
Abcam #16663
1:200

Suppl Fig 8

a

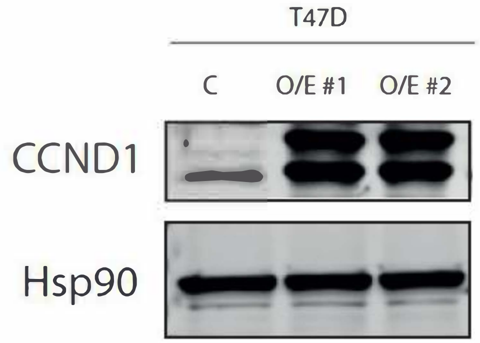

b

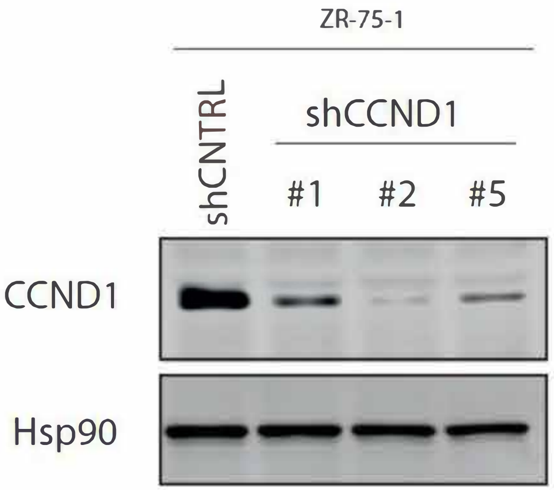

c

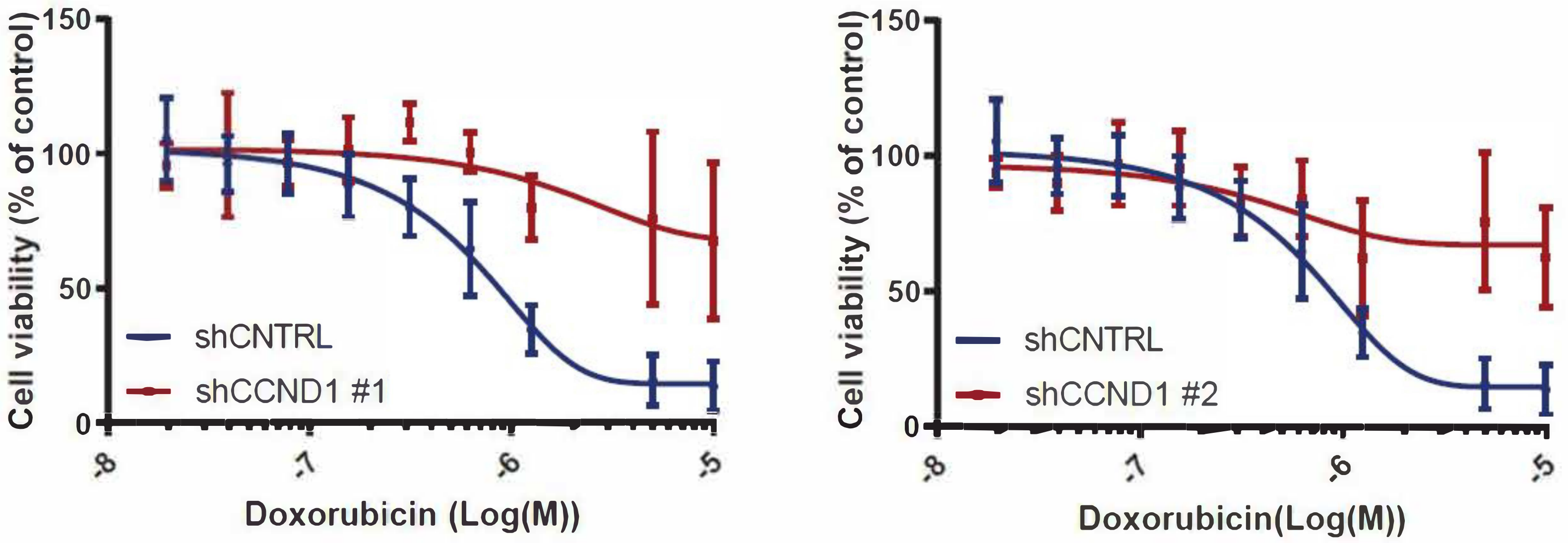

Supplement: Supplementary file 1 — Supplemental material [file 41523_2022_428_MOESM1_ESM.pdf]
