# Supplementary material for: Comprehensive characterization of pre- and post-treatment samples of breast cancer reveal potential mechanisms of chemotherapy resistance
Source: NPJ Breast Cancer. 2022 May 6;8:60. doi: 10.1038/s41523-022-00428-8 (PMC9076915; doi:10.1038/s41523-022-00428-8)
Supplement: Supplementary file 7 — Reporting Summary Checklist [file 41523_2022_428_MOESM7_ESM.pdf]

nature portfolio

Corresponding author(s): Esther H. Lips

Last updated by author(s): Dec 22, 2021

Reporting Summary

Nature Portfolio wishes to improve the reproducibility of the work that we publish. This form provides structure for consistency and transparency in reporting. For further information on Nature Portfolio policies, see our Editorial Policies and the Editorial Policy Checklist.

Statistics

For all statistical analyses, confirm that the following items are present in the figure legend, table legend, main text, or Methods section.

n/a	Confirmed	
☐	☒	The exact sample size (*n*) for each experimental group/condition, given as a discrete number and unit of measurement
☐	☒	A statement on whether measurements were taken from distinct samples or whether the same sample was measured repeatedly
☐	☒	The statistical test(s) used AND whether they are one- or two-sided<br>*Only common tests should be described solely by name; describe more complex techniques in the Methods section.*
☒	☐	A description of all covariates tested
☒	☐	A description of any assumptions or corrections, such as tests of normality and adjustment for multiple comparisons
☐	☒	A full description of the statistical parameters including central tendency (e.g. means) or other basic estimates (e.g. regression coefficient) AND variation (e.g. standard deviation) or associated estimates of uncertainty (e.g. confidence intervals)
☐	☒	For null hypothesis testing, the test statistic (e.g. *F*, *t*, *r*) with confidence intervals, effect sizes, degrees of freedom and *P* value noted<br>*Give P values as exact values whenever suitable.*
☒	☐	For Bayesian analysis, information on the choice of priors and Markov chain Monte Carlo settings
☒	☐	For hierarchical and complex designs, identification of the appropriate level for tests and full reporting of outcomes
☐	☒	Estimates of effect sizes (e.g. Cohen's *d*, Pearson's *r*), indicating how they were calculated

Our web collection on statistics for biologists contains articles on many of the points above.

Software and code

Policy information about availability of computer code

Data collection	Microsoft Access and Microsoft Excel were used to collect all clinical data and compute the NRI. Nanodrop and Agilent Bioanalyzer software was used to asses RNA and DNA quantity and quality, and sequence libraries. Sequencing was done on an Illumina 2500 (WES) or Illumina 2000 (RNAseq) with associated software.
Data analysis	WES: Sequencing reads were aligned using bwa [ref46]. Duplicate removal, base recalibration, variant calling and annotation was done using the GATK Haplotype caller[ref47], SNPEff[ref48] and SNPSift[ref49]. Somatic variants were identified using Strelka[ref50], annotated using the Ensembl Variant Effect Predictor[ref51] and genotyped per patient in exome sequencing and RNAseq data using samtools pileup[52] and custom perl scripts. Copy number estimations were generated using Cnvkit[ref53].<br><br>RNAseq: Reads were aligned to the human transcriptome (Homo_sapiens.GRCh37.75.gtf) using Tophat v2.1[ref55]. Readcounts per gene were calculated using lcount[ref56], and normalized using DESeq2[ref57]. The SVA R package[ref58] was used to combine and batch correct gene expression datasets. Gene expression levels were associated with chemotherapy response using samr[ref59]; differential gene expression between pre- and post-treatment samples was analyzed using DESeq2[ref57]. Subsequent pathway / GO-term enrichment was performed using the Reactome Cytoscape plugin[ref60, ref61] and geneset enrichment was performed using GSEA[ref62]. All downstream analysis (including correlation and Kaplan-Meier analyses) were done in R.

For manuscripts utilizing custom algorithms or software that are central to the research but not yet described in published literature, software must be made available to editors and reviewers. We strongly encourage code deposition in a community repository (e.g. GitHub). See the Nature Portfolio guidelines for submitting code & software for further information.

Data

Policy information about availability of data

All manuscripts must include a data availability statement. This statement should provide the following information, where applicable:

- Accession codes, unique identifiers, or web links for publicly available datasets
- A description of any restrictions on data availability
- For clinical datasets or third party data, please ensure that the statement adheres to our policy

The WES data is available through EGA: EGAS 0000100587 (dataset EGAD00001008442).
The microarray RNA data is available through GEO: GSE34138.
The RNAseq data is available through EGA (raw data): EGAS 00001005876 (EGAD00001008421 and EGAD00001008433) and GEO (processed data): GSE191127 and GSE192341.

Field-specific reporting

Please select the one below that is the best fit for your research. If you are not sure, read the appropriate sections before making your selection.

☒ Life sciences ☐ Behavioural & social sciences ☐ Ecological, evolutionary & environmental sciences

For a reference copy of the document with all sections, see nature.com/documents/nr-reporting-summary-flat.pdf

Life sciences study design

All studies must disclose on these points even when the disclosure is negative.

Sample size	For the pre-treatment RNAseq data analysis we collected a large cohort of ER+ and TN breast cancer. We collected consecutively during the years 2000-2013 a biopsy of all breast cancer patients in the NKI-AVL. We tried to collect a large dataset, to capture intra tumor heterogeneity.<br><br>For the pre-post chemotherapy dataset we had to rely on the institute's biobank and the precence of a substantial amount of tumor tissue post-neoadjuvant treatment. Consequently the number is small with n=22 samples. However this number is in line with published literature.
Data exclusions	For the analysis of matched samples before and after treatment, we used the following additional selection criteria: 1) No pCR after receiving standard of care neoadjuvant chemotherapy; 2) > 50% tumor cells in all samples; 3) Availability of fresh frozen material of all samples; and 4) Availability of matched blood.
Replication	*Describe the measures taken to verify the reproducibility of the experimental findings. If all attempts at replication were successful, confirm this OR if there are any findings that were not replicated or cannot be reproduced, note this and describe why.*
Randomization	*Describe how samples/organisms/participants were allocated into experimental groups. If allocation was not random, describe how covariates were controlled OR if this is not relevant to your study, explain why.*
Blinding	*Describe whether the investigators were blinded to group allocation during data collection and/or analysis. If blinding was not possible, describe why OR explain why blinding was not relevant to your study.*

Reporting for specific materials, systems and methods

We require information from authors about some types of materials, experimental systems and methods used in many studies. Here, indicate whether each material, system or method listed is relevant to your study. If you are not sure if a list item applies to your research, read the appropriate section before selecting a response.

Materials & experimental systems

n/a	Involved in the study	
☐	☒	Antibodies
☐	☒	Eukaryotic cell lines
☒	☐	Palaeontology and archaeology
☒	☐	Animals and other organisms
☒	☐	Human research participants
☐	☒	Clinical data
☒	☐	Dual use research of concern

Methods

n/a	Involved in the study	
☒	☐	ChIP-seq
☒	☐	Flow cytometry
☒	☐	MRI-based neuroimaging

Antibodies

Antibodies used

the following antibodies were used: rabbit anti-p27 Kip1 (Cell Signaling 2552, 1:1000); rabbit anti-Cyclin D1 (Abcam 16663, 1:200) i; beta-catenin (Cell signaling #8480, 1:2000); CCND1 (ab239794, 1:1000); Hsp90 (sc-7947,1:1000).

Validation

Antibodies were used according to the manufacturer' datasheet

Eukaryotic cell lines

Policy information about cell lines

Cell line source(s)

293T, T47D, MDA-MB-415, and ZR-75 cell lines from ATCC.

Authentication

All cell lines have been genotyped by Eurofins.

Mycoplasma contamination

All cell lines tested negative for mycoplasma contamination.

Commonly misidentified lines
(See ICLAC register)

N/S

Clinical data

Policy information about clinical studies

All manuscripts should comply with the ICMJE guidelines for publication of clinical research and a completed CONSORT checklist must be included with all submissions.

Clinical trial registration

NCT00448266, NCT01057069

Study protocol

https://clinicaltrials.gov

Data collection

Biopsies of primary breast tumors were collected and snap-frozen in liquid nitrogen prior to treatment from women with locally advanced breast cancer at the Netherlands Cancer Institute between 2000 and 2013. All patients had received neoadjuvant treatment as part of ongoing clinical trials (NCT00448266, NCT01057069), or were treated off protocol according to the standard arms of one of these studies.

Outcomes

Primary outcome was pCR and NRI; secondary outcome was recurrence free survival.
